# Long non‐coding RNA LINC00673 silencing inhibits proliferation and drug resistance of prostate cancer cells via decreasing KLF4 promoter methylation

**DOI:** 10.1111/jcmm.14883

**Published:** 2019-12-27

**Authors:** Zhenming Jiang, Yuxi Zhang, Xi Chen, Pingeng Wu, Dong Chen

**Affiliations:** ^1^ Department of Urology The First Hospital of China Medical University Shenyang China; ^2^ Department of Urology People’s Hospital of Datong Hui and Tu Autonomous County Xining China; ^3^ Department of Pharmacy The First Hospital of China Medical University Shenyang China; ^4^ Central Lab The First Hospital of China Medical University Shenyang China

**Keywords:** drug resistance, Kruppel‐like factor 4, LINC00673, methylation, proliferation, prostate cancer

## Abstract

Prostate cancer is one of the major causes of cancer‐related mortality in men across the world. Recently, long non‐coding RNAs (lncRNAs) and Kruppel‐like factor 4 (KLF4) have been reported to participate in the biology of multiple cancers including prostate cancer. Here, this study aimed to explore the possible role of LINC00673 in prostate cancer via KLF4 gene promoter methylation. Microarray‐based gene expression profiling of prostate cancer was employed to identify differentially expressed lncRNAs and genes, after which the expression of LINC00673 and KLF4 in prostate cancer tissues was determined using RT‐qPCR. Next, the relationship between LINC00673 and KLF4 was evaluated using in silico analysis. Further, the effect of LINC00673 and KLF4 on cell proliferation and drug resistance of transfected cells was examined with gain‐ and loss‐of‐function experimentation. It was found that LINC00673 was highly expressed, while KLF4 was poorly expressed in prostate cancer tissues. Additionally, LINC00673 could bind to KLF4 gene promoter region and recruit methyltransferase to the KLF4 gene promoter region. Moreover, LINC00673 silencing was demonstrated to reduce methylation of the KLF4 gene promoter to elevate the expression of KLF4, thus suppressing the proliferation and drug resistance of prostate cancer cells. In summary, LINC00673 silencing could drive demethylation of the KLF4 gene promoter and thus inhibit the proliferation and drug resistance of prostate cancer cells, suggesting that silencing of LINC00673 and elevation of KLF4 could serve as tumour suppressors in prostate cancer.

## 1 INTRODUCTION

As a non‐skin tumour accompanied by variable natural history, prostate cancer is a highly prevalent malignancy and represents one of the leading causes of cancer‐related deaths in the male populace.^1^, ^2^ Risk factors responsible for prostate cancer comprise smoking, alcohol consumption and levels of male androgens.^3^ Paclitaxel is widely regarded as an essential important drug for prostate cancer treatment; however, paclitaxel resistance ensues in many prostate cancer patients, which highlights the significance of developing strategies to inhibit drug resistance.^4^ Moreover, owing to androgen deprivation treatment therapy and increased doses of radiation, great advances have been achieved in the prognosis of prostate cancer patients; however, numerous patients still suffer from recurrence.^5^ Hence, the importance of developing new biological targets to improve the treatment outcomes of prostate cancer cannot be overestimated.

Long non‐coding RNAs (lncRNAs) are a family of transcripts that are 200 nucleotides long, and their differential expressions are closely implicated in various cellular processes of malignancies such as invasion, apoptosis or proliferation.^6^ Microarray‐based gene expression profiling analyses in the present study revealed that LINC00673 was differentially expressed in prostate cancer. Furthermore, differential expressions of LINC00673 have been documented in numerous cancers. For example, LINC00673 is overexpressed in both hepatocellular carcinoma and tongue squamous cell carcinoma (TSCC) and also associated with poor prognoses of these carcinomas.^7^, ^8^ In addition, Ba et al suggested that LINC00673 could potentiate the progression of gastric cancer, partly owing to its suppressive roles in the expression of Kruppel‐like factor 4 (KLF4).^9^ KLF4 belongs to the KLFs class of transcriptional mediators which can bind to DNA and are widely expressed in human cancers serving as oncogenes or tumour suppressors.^10^ KLF4 is dysregulated in various cancers including prostate cancer, and its enforced expression has been confirmed to attenuate cell metastasis and growth, thus functioning as an inhibitor of prostate cancer.^11^ Additionally, the expression of KLF4 stimulated by lysophosphatidic acid has been revealed to be involved in the proliferation and migration abilities of prostate cancer cells.^12^ DNA methylation is closely linked to numerous human cancers and KLF4, as readers of DNA methylation specific to sequence, also requires methylated cytosine‐phosphate‐guanine (CpG).^13^, ^14^ Based on these findings, we hypothesized that LINC00673 could participate in the progression of prostate cancer via interaction with KLF4. Therefore, the current study aims to investigate the underlying mechanism by which LINC00673 regulates the development of prostate cancer in regard with KLF4 involvement.

## 2 MATERIALS AND METHODS

### 2.1 Ethics statement

The current study was approved by the Ethics Committee and Experimental Animal Ethics Committee of The First Hospital of China Medical University. Signed informed consents were obtained from all participants or their legal guardians prior to the experiment. All animal experimentation strictly adhered to principles aiming to minimize the number, suffering and discomfort of the included animals.

### 2.2 Microarray‐based gene expression profiling

Prostate cancer‐related microarray data were retrieved from the Gene Expression Omnibus (GEO) database (https://www.ncbi.nlm.nih.gov/geo/). The obtained data were subjected to standardized pre‐treatment with the “Affy” package of R Language Programming.^15^ Next, the “Limma” package was employed to screen the differentially expressed genes (DEGs)^16^ with |log2FC|> 1.5 and *adj.P Val (P* value after correction*)* <.05 serving as the threshold. Subsequently, a heat map of the obtained DEGs was plotted.

### 2.3 Study subjects

Prostate cancer cells (PC3, LNCap and DU145), paclitaxel‐resistant cell line (DU145/pr) and normal prostate epithelial cell line (RWPE‐1) were all purchased from Cell Resource Center of Shanghai Institutes for Biological Sciences, Chinese Academy of Sciences .

Additionally, prostate cancer tissues were collected from 48 patients who underwent radical prostatectomy at The First Hospital of China Medical University between January 2015 and August 2017. All the included patients were aged between 55 and 84 years old with an average age of 69 years and did not undergo drug therapy and radiotherapy prior to the experiment. Among these patients, 15 patients were at the T1 stage, 15 at the T2 stage and 18 at the T3 stage. A portion of the prostate cancer tissues and adjacent normal tissues were cryopreserved at −80°C, and others were fixed using 10% formalin, dehydrated, paraffin‐embedded and stored for subsequent experimentation.

### 2.4 In situ hybridization

Tissue sections were attached to slides pre‐treated with 10% polylysine to perform in situ hybridization in accordance with the instructions of the kits (BOSTER Biological Technology Co., Ltd.). Next, the sections were hybridized with digoxin‐labelled LINC00673 probe (Exiqon) at a constant temperature of 52°C for 16 hours, warm‐bathed with biotinylated mouse anti‐digoxin at 37°C for 60 minutes and incubated with streptavidin biotin peroxidase complex (SABC), followed by diaminobenzidine (DAB) developing. The obtained results were independently scored by two pathologists. The cells presenting with tan‐stained nuclei were regarded as the positive cells. A total of five visual fields were randomly selected from each section under a 200‐fold microscope to calculate the percentage of positive cells. The percentage of the positive cells <5% was indicative of negative cells, while that ≥5% was indicative of positive cells.

### 2.5 Cell culture and treatment

A total of 10 μg lentiviral vector Pcdh of target plasmid, 7.5 μg helper plasmid PAX and 5 μg helper plasmid Pmd2G were, respectively, diluted with 750 μL of opti‐MEM (Gibco) and allowed to stand for 5 minutes. Separately, 112.5 μg PEI was diluted with 750 μL opti‐MEM and allowed to stand at room temperature for 5 minutes. Subsequently, the two aforementioned solutions were mixed uniformly. After 20 minutes, the mixture was added to the corresponding cell culture dishes and cultured with 5% CO_2_ in air at 37°C with the medium renewed after 6 hours. After 48 hours, the cell supernatant was collected. Following 24‐hour culture with 8 mL of complete medium, the cell supernatant was collected. A total of 1 × 10^5^ cells were treated with lentivirus and cultured with the medium for 24 hours. Subsequently, the fluorescence intensity was detected using a fluorescence microscope. Next, the cells were selected for monoclonal cultivation to obtain stable cell lines for xenograft tumour in nude mice. All the following plasmids were purchased from Dharmacon: small interfering RNA (si)‐negative control (NC), si‐LINC00673, pcDNA‐NC, pcDNA‐LINC00673 and pcDNA‐KLF4.

### 2.6 Reverse transcription quantitative polymerase chain reaction (RT‐qPCR)

Total RNA content extraction from the cells was performed with the Trizol method (15596026; Invitrogen). The integrity of the extracted RNA was then identified using 1% agarose gel electrophoresis, and RNA concentration and purity were measured using a NanoDrop ND‐1000 spectrophotometer. Subsequently, the RNA was reverse transcribed into complementary DNA (cDNA) according to the instructions of the PrimeScript RT reagent kits (RR047A; Takara). All the primers (Table 1) were synthesized by Beijing Genomics Institute Biotech Co., Ltd. β‐actin was regarded as the internal control for LINC00673 and mRNA. The fold changes were calculated by means of relative quantification (the $2^{-\Delta \Delta C_{t}}$ method).

**Table 1:** Primer sequences for reverse transcription quantitative polymerase chain reaction

Gene	Primer sequence (5′‐3′)
LINC00673	Forward: TCAGAAGACCCACGACCTCT
	Reverse: AATACCTCCAGCTTGGCGAC
KLF4	Forward: GCCCCTCGGGCGGCTTCGT GGCCGAGCTC
	Reverse: CGTACTCGCTGCCAGGG GCG
Cyclin D2	Forward: GAGGGTCATATTCTACCAGG
	Reverse: GAACCCTCAAAACCCACGGATT
Cyclin B1	Forward: AGGTTGTTGCAGGAGACCAT
	Reverse: CAGGTGCTGCATAACTGGAA
β‐actin	Forward: GCAAAGACCTGTACGCCAACA
	Reverse: TGCATCCTGTCGGCAATG

Abbreviation: KLF4, Kruppel‐like factor 4.

### 2.7 Western blot analysis

The cells in each group were lysed with 500 μL of radioimmunoprecipitation assay (RIPA; Pierce). Protein concentration was then measured using bicinchoninic acid (BCA) protein assay kits (BCA1‐1KT; Sigma‐Aldrich). Afterwards, the total proteins extracted from the cells were subjected to sodium dodecyl sulphate‐polyacrylamide gel electrophoresis (SDS‐PAGE). Subsequently, the proteins were transferred onto membranes, which were blocked with 5% skimmed milk‐Tris‐buffered saline Tween‐20 (TBST) for 1 hour. With glyceraldehyde‐3‐phosphate dehydrogenase (GAPDH) serving as the internal control, the membrane was incubated overnight at 4°C with rabbit polyclonal antibodies against KLF4 (dilution ratio of 1:1000, ab106629), Cyclin D2 (dilution ratio of 1:1000, ab207604) and Cyclin B1 (dilution ratio of 1:50 000, ab32053). All the aforementioned antibodies were purchased from Abcam Inc. Following incubation with the corresponding secondary antibody immunoglobulin G (IgG; A21020; Abbkine) at 37°C for 45 minutes, the membranes were rinsed with TBST for 45 minutes and allowed to react with the enhanced chemiluminescence reagent (ECL; ECL808‐25; Biomiga) for 1 minute. The protein grey values were analysed using the Gel‐Pro Analyzer 4.0 (Media Cybernetics) to determine the protein expression.

### 2.8 3‐(4,5‐dimethylthiazol‐2‐yl)‐2, 5‐diphenyltetrazolium bromide (MTT) assay

After 48 hours of conventional treatment, the cells were cultured with Roswell Park Memorial Institute (RPMI) 1640 medium containing 10% foetal bovine serum (FBS) and then dispersed into a cell suspension. After counting, the cells were inoculated in a 96‐well plate at a density of 8 × 10^3^ cells/well. After incubation at 37°C with 5% CO_2_ in air for 24 hours, the cells were administered paclitaxel until the final concentrations reached specific parameters of 0, 20, 40 and 60 nmol/L. After 24 hours, cells in each well were added with 10 μL MTT solution (5 mg/mL) and incubated at 37°C for 4 hours. Afterwards, the cells were added with 100 μL dimethyl sulphoxide (DMSO) and oscillated for 10 minutes to uniformly dissolve the crystals. The optical density (OD) value was measured in each well at a wavelength of 570 nm using a microplate reader to calculate the cell inhibition rate (IR; %). Subsequently, the IR curve was plotted, and the half maximal inhibitory concentration (IC50) was calculated.

### 2.9 Fluorescence in situ hybridization (FISH)

The subcellular localization of LINC00673 in DU145 cells was identified using FISH kits according to the instructions of the Ribo™ lncRNA FISH probe Mix (RiboBio Company). In brief, DU145 cells were inoculated onto the cover glass in a 6‐well plate at a density of 6 × 10^4^ cells/well. When cell confluence approximately reached 80% after culture for 1 day, the cover glass was rinsed with phosphate buffered saline (PBS), fixed with 1 mL 4% paraformaldehyde at room temperature and treated with protease K (2 μg/mL), glycine and acetylation reagent. Following incubation with 250 μL of pre‐hybridization solution and 250 μL of hybridization solution containing probe‐labelled LINC00673 (300 ng/mL), the cover glass was rinsed with phosphate buffered saline with Tween‐20 (PBST), and the nucleus was stained with 4′,6‐diamidino‐2‐phenylindole (DAPI; 1:800) diluted with PBST. Subsequently, the glass was added to the 24‐well plate for staining and then rinsed with PBST. Finally, the cover glass was mounted with the anti‐fluorescence quenching agent. Images were acquired from five randomly selected visual fields under a fluorescence microscope (Olympus).

### 2.10 Dual‐luciferase reporter gene assay

Firstly, we designed the dual‐luciferase reporter plasmid of KLF4 target gene promoter containing KLF4 wild‐type (KLF4‐Wt) and KLF4 mutated at the putative LINC00673 binding sites (KLF4‐mutant type [Mut]). The sequence of KLF4‐Wt was TGAGGGGTGGGGGGCGCTAGGGGTGTGGAGAGGAGGCAGTGCCCAGCACTGTGCAGCGTGAACTGGGAGCCTCAAG and that of KLF4‐Mut was CTCTGGAGTTTGGTACTGAGAAACATAGAGAGGCGTGAGACTTTAGCAATCCACATAGTGCCGTTGCTCCCGGGCG. Subsequently, KLF4‐Wt and KLF4‐Mut were co‐transfected with overexpressing LINC00673 and NC plasmids into DU145 cells, respectively. After 24 hours of treatment, the cells were lysed and centrifuged at 12 000 r/min for 1 minute, after which, the supernatant was collected. The luciferase activity was then detected using a Dual‐Luciferase^®^ Reporter Assay System (E1910; Promega). Each cell sample was added with 100 μL of firefly luciferase reagent to detect the firefly luciferase. The relative luciferase activity was expressed as the ratio of firefly luciferase to renilla luciferase.

### 2.11 Chromatin immunoprecipitation (ChIP)

ChIP kits (Millipore Inc) were employed in order to determine the enrichment of DNA methyltransferase (DNMT)1, DNMT3a and DNMT3b in the KLF4 gene promoter region. When cell confluence approximately reached 70%‐80%, the cells were fixed with 1% formaldehyde at room temperature for 10 minutes to cross‐link the intracellular DNA and protein. Next, the cross‐linked DNA and protein were randomly cracked into fragments, which were centrifuged at 13 000 r/min at 4°C. The collected supernatant was then sub‐packed into three tubes and incubated overnight at 4°C with positive control antibody RNA polymerase Ⅱ, NC antibody IgG from normal mice, and specific rabbit antibodies of target proteins (DNMT1 [Abcam Inc, ab13537], DNMT3a [Abcam Inc, ab2850] and DNMT3b [Abcam Inc, ab2851]). Subsequently, protein agarose/sepharose was applied to precipitate the endogenous DNA‐protein complex. After centrifugation, the supernatant was discarded, and the non‐specific complex was washed and de‐cross‐linked overnight at 65°C. The obtained DNA fragments were extracted and purified using phenol/chloroform. The binding of KLF4 gene promoter region to DNMT1, DNMT3a and DNMT3b was finally detected using specific primers of the KLF4 gene promoter region.

### 2.12 RNA‐binding protein immunoprecipitation (RIP) assay

A RIP assay was performed in accordance with the instructions of the Magna RIP RNA‐Binding Protein Immunoprecipitation kits (Millipore Inc). Briefly, the cells were rinsed with pre‐cooled PBS, lysed for 30 minutes with 100 μL lysis buffer supplemented with protease inhibitor and ribonuclease inhibitor, and then centrifuged at 12 000 r/min at 4°C for 3 minutes. A small amount of supernatant was taken as the input positive control and added with 1 μg of the corresponding antibody. The remaining supernatant was added with NC antibody IgG from normal cells, specific rabbit antibodies of target protein (DNMT1 [Abcam Inc, ab13537], DNMT3a [Abcam Inc, ab2850], DNMT3b [Abcam Inc, ab2851]) and 10‐50 μL protein A/G‐beads. Next, the supernatant was incubated at 4°C overnight, centrifuged at 3000 r/min for 5 minutes, washed with 1 mL lysis buffer and precipitated using protein A/G‐beads, followed by centrifugation at 1000 r/min at 4°C for 1 minute. After being added with 15 μL of 2 × SDS loading buffer, the supernatant was heated for 10 minutes. RNA content was extracted and purified from the precipitate using a RNA extraction method. Finally, RT‐qPCR was conducted using specific primers of LINC00673 to verify the interaction between LINC00673 and DNMT1, DNMT3a and DNMT3b.

### 2.13 Bisulphite sequencing polymerase chain reaction (BSP) and methylation specific PCR (MSP)

Firstly, the cellular genome DNA was extracted, and Methyl Detector TM Bisulphite Modification Kits (Active Motif) were used for DNA methylation modification to perform PCR amplification. The sequence of KLF4 promoter was as follows: forward primer: 5′‐GGATTTGTTTTTTTATTT‐3′; reverse primer: 5′‐AATCAAAAAAAAAATATTCTCC‐3′. M band amplification indicated methylation (+) while U band amplification indicated unmethylation (−). BSP results were judged as follows: >50% indicated positive methylation (+), while <10% suggested that methylation was negative (−).

### 2.14 5‐ethynyl‐2′‐deoxyuridine (EdU) assay

Cells were inoculated in a 96‐well plate at a density of 1.6 × 10^5^ cells/well and cultured for 48 hours. After culture, EdU assay was performed in accordance with the protocols of EdU kits (C10310; Guangzhou RiboBio Co., Ltd.). Cells in each well were cultured with 100 μL of 50 μmol/L EdU at 37°C for 4 hours, fixed with 4% formaldehyde at room temperature for 15 minutes and treated with 0.5% Triton X‐100 at room temperature for 20 minutes for permeabilization. After that, cells in each well were incubated with 100 μL of Apollo^®^ compound (C10338‐2; RiboBio Company) at room temperature for 30 minutes, stained using 100 μL of hoechst33342 (Ribobio Company) for 30 minutes and observed under a fluorescence microscope (Olympus). The number of EdU positive cells (red blood cells) was calculated using the Image‐Pro Plus 6.0 software (Media Cybernetics).^17^

### 2.15 Flow cytometry

Cells undergoing different treatments were detached with 0.25% trypsin and dispersed into a single cell suspension. After being rinsed with PBS, the cells were centrifuged, fixed with 70% pre‐cooled ethanol at overnight 4°C and resuspended. Following centrifugation and pre‐cooled PBS rinsing, the cells were resuspended in 100 µL PBS, added with RNase until the final concentration reached 50‐70 µg/mL, and water‐bathed at 37°C for 30 minutes. When the final concentration reached 50 µg/mL after the addition of propidium iodide (PI), the cells were stained avoiding exposure to light at 4°C for 40 minutes and rinsed with PBS. The DNA content was then calculated using a 575 nm bandpass filter to calculate the percentage of cell cycle.

### 2.16 Xenograft tumour in nude mice

Cells undergone different treatments were washed with normal saline, detached with 1 mL 0.25% trypsin and incubated at 37°C. Detachment was terminated with the addition of 3 mL complete medium, after which the cells were centrifuged and the cell precipitate was collected. Cells were then dispersed into a single cell suspension and counted. A total of 2 × 10^6^ cells were resuspended with 50 μL normal saline, mixed with 50 μL Matrigel, and then subcutaneously injected on the flank of nude mice (Shanghai Experimental Animal Center, Chinese Academy of Sciences). The tumour volume was measured and recorded after a week of inoculation. When the mean volume reached 100 mm^3^ at the 3rd week, the mice were intraperitoneally injected with paclitaxel (50 μg/kg; 0.01 mL/g) once every 2 days for a total of seven times. Finally, the tumour volumes were measured twice a week.

### 2.17 Statistical analysis

Statistical analyses were performed using the SPSS21.0 software (IBM Corp). Measurement data were presented as mean ± standard deviation (SD). Data between two groups were analysed using the independent sample *t* test and corrected using Welch. The normality test of data among multiple groups was analysed using the Shapiro–Wilk method. Data conforming to normal distribution were analysed using one‐way analysis of variance (ANOVA). Pairwise comparisons of data among multiple data were conducted using the least significant difference (LSD) *t* test. Data presenting with skew distribution were compared using the non‐parametric Kruskal–Wallis test. Proliferative activities and tumour volumes at different time‐points were analysed using repeated measures ANOVA. A value of *P* < .05 indicated statistical significance.

## 3 RESULTS

### 3.1 LINC00673 is highly expressed while KLF4 is poorly expressed in prostate cancer

**Figure 1:**
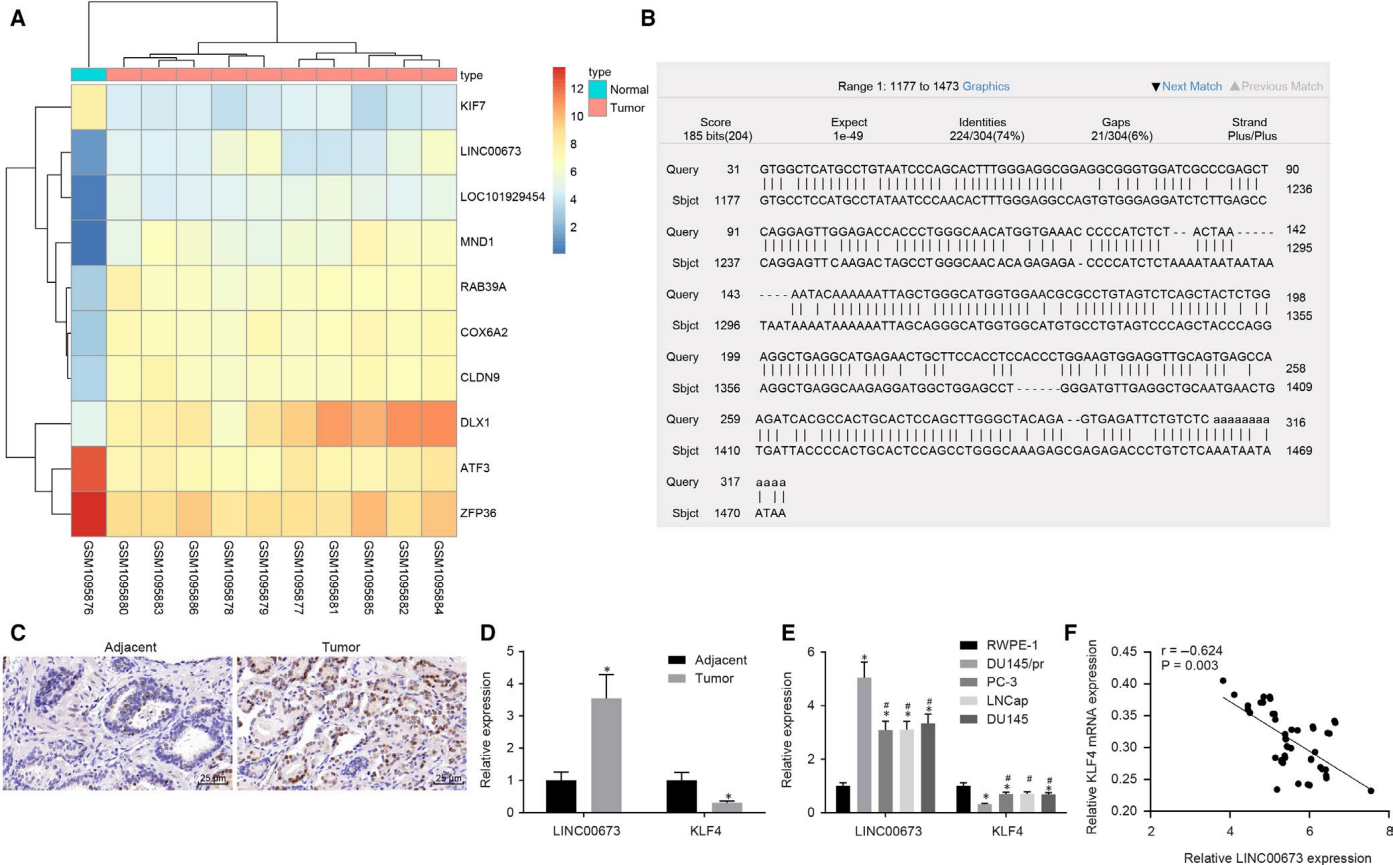
Highly expressed LINC00673 and poorly expressed KLF4 are evident in prostate cancer. A, A heat map of LINC00673 expression in adjacent normal tissues and prostate cancer tissues retrieved from GSE45016. B, Promoter regions of LINC00673 and KLF4 gene. C, Expression of LINC00673 in prostate cancer tissues and adjacent normal tissues detected by FISH (×400). D, Expressions of LINC00673 and KLF4 in prostate cancer tissues and adjacent normal tissues measured by RT‐qPCR. E, Expressions of LINC00673 and KLF4 in prostate cancer cells (PC3, LNCap and DU145), paclitaxel‐resistant cell line (DU145/pr) and normal prostate epithelial cell line (RWPE‐1) determined by RT‐qPCR. F, Correlation analysis of KLF4 and LINC00673 expression. * *P* < .05, vs adjacent normal tissues/RWPE‐1 cell line; # *P* < .05, vs DU145/pr cell. Data (mean ± SD) between two groups were compared using the unpaired t test, and data among multiple groups were compared using one‐way ANOVA. n = 48

Initially, we performed a differential gene expression analysis on the prostate cancer‐related expression data set, GSE45016. A heat map illustrating the most differentially expressed genes is shown in Figure 1A. Subsequently, higher expressions of LINC00673 were found in prostate cancer tissues relative to adjacent normal tissues (*P* < .05). Ensuing results from the BLAST website (https://blast.ncbi.nlm.nih.gov/Blast.cgi?PROGRAM=blastn%26PAGE_TYPE=BlastSearch%26BLAST_SPEC=%26LINK_LOC=blasttab%26LAST_PAGE=tblastn) further revealed that the promoters of LINC00673 and KLF4 possess the same base sequence (Figure 1B). Additionally, the expression of LINC00673 was detected in prostate cancer tissues using FISH, and the results revealed that LINC00673 was highly expressed in prostate cancer tissues as compared with adjacent normal tissues (*P* < .05; Figure 1C). Furthermore, RT‐qPCR detected that the expression of LINC00673 was higher while that of KLF4 was lower in prostate cancer tissues when compared with adjacent normal tissues (both *P* < .05; Figure 1D). In addition, RT‐qPCR was applied to detect the expression of LINC00673 and KLF4 in prostate cancer cells (PC3, LNCap and DU145), paclitaxel‐resistant cell line (DU145/pr) and normal prostate epithelial cell line (RWPE‐1). The results demonstrated that LINC00673 was highly expressed while KLF4 was poorly expressed in other cells when compared with RWPE‐1 cells; the expression of LINC00673 was higher while that of KLF4 was lower in PC3, LNCap and DU145 cells compared with DU145/pr cells (all *P* < .05; Figure 1E). Correlation analysis results further revealed that KLF4 was negatively correlated with LINC00673 (Figure 1F). In addition, as shown in Table 2, LINC00673 expression was found to be associated with tumour size, tumour node metastasis (TNM) stage and lymph node metastasis (LNM), while no correlations were found in regard to the age of patients with prostate cancer. All these results verified that prostate cancer exhibits high expression of LINC00673 and low expression of KLF4.

**Table 2:** Correlation of LINC00673 expression with the clinical characteristics of patients with prostate cancer

Clinical characteristics	Cases	LINC00673	*P*
	(n)	(mean ± SD)	
Age (years)			
≤69	26	3.430 ± 0.711	.231
>69	22	3.688 ± 0.760	
Tumour size (cm)			
≤3	20	3.060 ± 0.588	<.0001
>3	28	3.897 ± 0.632	
Tumour node metastasis stage			
T1	15	2.980 ± 0.663	<.0001
T2	15	3.448 ± 0.435	
T3	18	4.104 ± 0.602	
Lymph node metastasis			
Yes	26	3.144 ± 0.622	.0002
No	22	3.890 ± 0.656	

Abbreviation: SD, standard deviation.

### 3.2 LINC00673 silencing suppresses proliferation in prostate cancer cells

**Figure 2:**
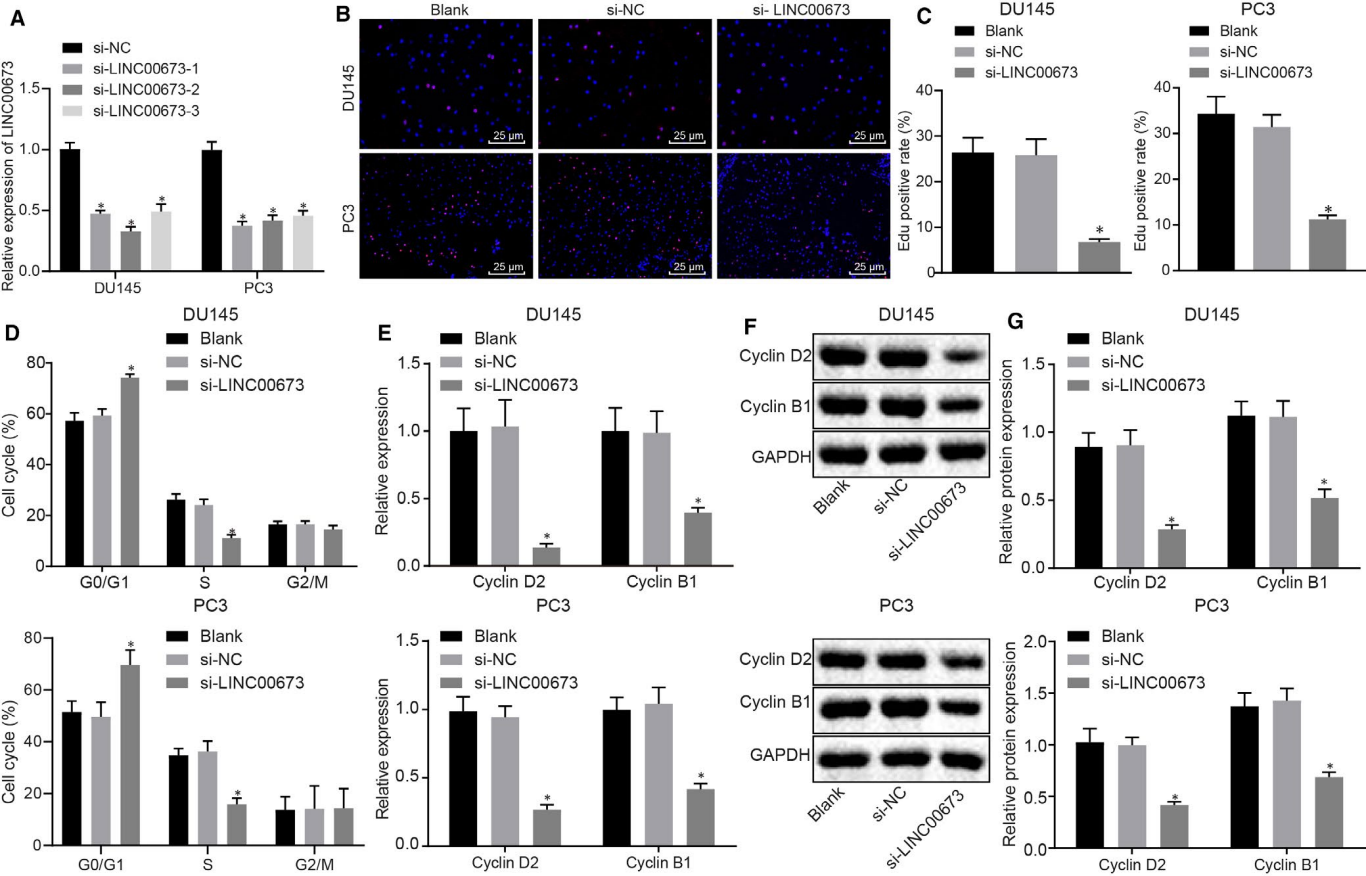
LINC00673 knockdown represses prostate cancer cell proliferation. A, Silencing efficiency of LINC00673 in DU145 and PC3 cell lines detected using RT‐qPCR. B, C, Cell proliferation in DU145 and PC3 cells following different treatments detected by EdU assay (×400). D, Cell cycle of DU145 and PC3 cells following different treatments determined by flow cytometry. E, mRNA expression of Cyclin D2 and Cyclin B1 in DU145 and PC3 cells following different treatments determined by RT‐qPCR. F and G, Western blot analysis of Cyclin D2 and Cyclin B1 proteins in DU145 and PC3 cells following different treatments. * *P* < .05, vs DU145 and PC3 cells without treatment. Data (mean ± SD) among multiple groups were compared using one‐way ANOVA. The experiment was repeated in triplicate to obtain the mean value

After elucidating the expression patterns of LINC00673 and LINC00673, we treated the prostate cancer cell lines DU145 and PC3 with si‐NC and si‐LINC00673 to conduct a series of assays to verify the effects of LINC00673 on prostate cancer cell proliferation. Initially, we attenuated LINC00673 in the two aforementioned cell lines and selected the most efficient sequence for subsequent experimentation (Figure 2A). EdU assay was then performed to detect cell proliferation which revealed that EdU positive cells were stained with red colouration (Figure 2B,C). Compared with cells without treatment, proliferative rate was reduced in DU145 cells treated with si‐LINC00673 (*P* < .05), but no significant differences were detected in DU145 cells treated with si‐NC (*P* > .05). Subsequently, cell cycle was measured using flow cytometry, and the results revealed that si‐LINC00673‐treated DU145 cells exhibited increased proportion arrested at the G0/G1 phase but decreased proportion arrested at the S phase when compared with the DU145 cells without treatment (*P* < .05); however, the cell cycle did not differ in si‐NC‐treated cells (*P* > .05; Figure 2D). Next, the effects of LINC00673 on the mRNA and protein expression of Cyclin D2 and Cyclin B1 were determined using RT‐qPCR and Western blot analysis. The results (Figure 2E,G) demonstrated that the mRNA and protein expressions of Cyclin D2 and Cyclin B1 were down‐regulated in DU145 cells following si‐LINC00673 treatment when compared with DU145 cells without treatment (*P* < .05), while no significant differences were observed in DU145 cells following si‐NC treatment (*P* > .05). Additionally, we repeated all the above‐mentioned experiments in PC3 cells. The findings verified that all the results obtained from DU145 cell lines were in accordance with the PC3 cell lines. The aforementioned findings demonstrated that prostate cancer cell proliferation can be inhibited by LINC00673 silencing.

### 3.3 LINC00673 silencing inhibits drug resistance of prostate cancer cells

**Figure 3:**
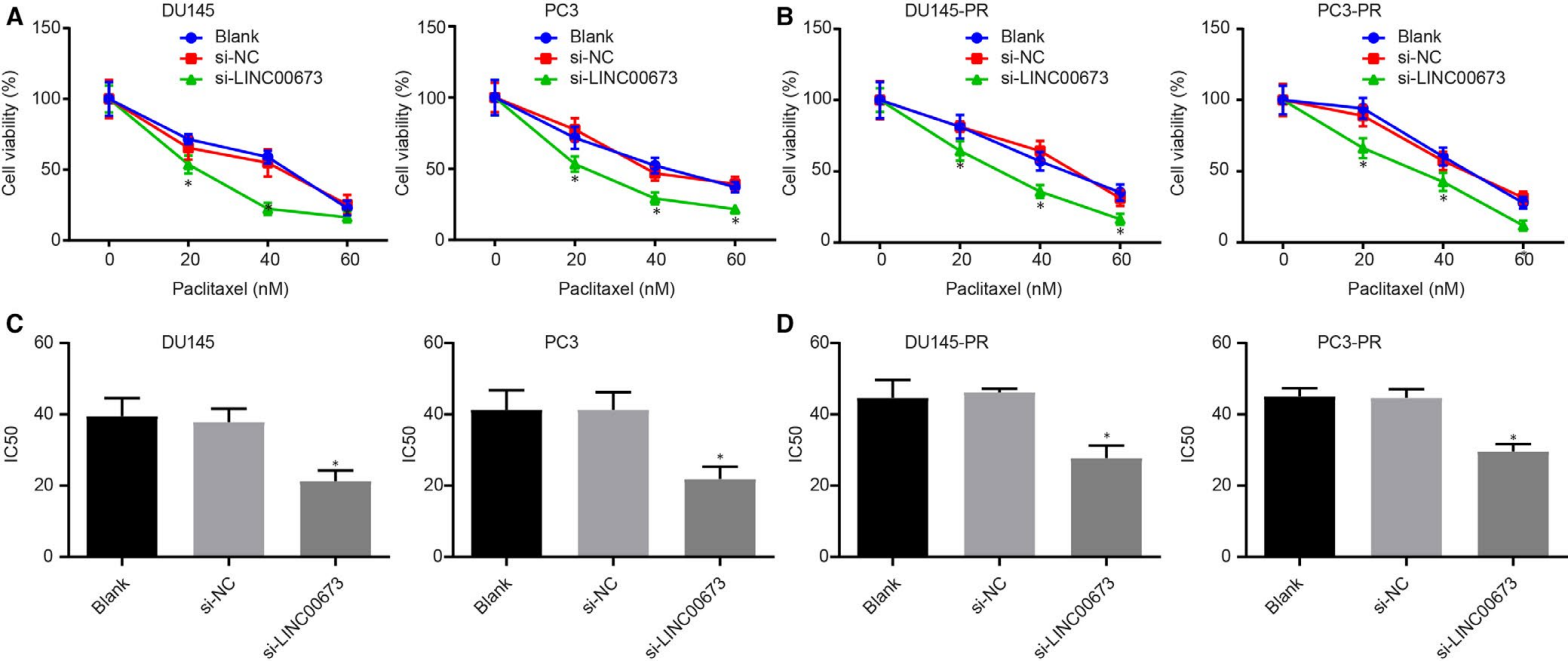
LINC00673 knockdown curtails drug resistance of prostate cancer cells. A, Proliferation of DU145 and PC3 cells treated with paclitaxel at various concentrations detected by MTT assay. B, Proliferation of paclitaxel‐resistant DU145 and PC3 cells measured by MTT assay. C, IC50 value of DU145 and PC3 cells treated with paclitaxel at various concentrations measured by MTT assay. D, IC50 value of docetaxel‐resistant DU145 and PC3 cells measured by MTT assay. *P < .05, vs DU145 and PC3 cells without treatment. Data (mean ± SD) among multiple groups were compared using repeated measures ANOVA. The experiment was repeated in triplicate to obtain the mean value

After LINC00673 silencing was found to be able to inhibit prostate cancer cell proliferation, the focus of the current study was shifted to determine the effects of LINC00673 on drug resistance of prostate cancer cells with DU145 and paclitaxel‐resistant DU145 cells receiving si‐NC or si‐LINC00673 treatment. Cell proliferation detected using MTT assay (Figure 3A,B) revealed that after paclitaxel treatment with different concentrations at the 5th day of culture, DU145 and paclitaxel‐resistant DU145 cells treated with si‐LINC00673 exhibited reduced proliferation ability (*P* < .05), while DU145 and paclitaxel‐resistant DU145 cells treated with si‐NC showed no differences when compared with DU145 and paclitaxel‐resistant DU145 cells without treatment (*P* > .05). Following paclitaxel treatment, the IC50 value was determined to be decreased in si‐LINC00673‐treated paclitaxel‐resistant DU145 and PC3 cells (*P* < .05), but did not differ in si‐NC‐treated DU145 cells when compared with that in paclitaxel‐resistant DU145 and PC3 cells without treatment (*P* > .05). Similar trends were also observed in PC3 cells confirming our findings (*P* < .05; Figure 3C). Moreover, we detected IC50 values in DU145 and PC3 cells treated with docetaxel at various concentrations using MTT assay and observed a reduced IC50 value in si‐LINC00673‐treated DU145 and PC3 docetaxel‐resistant cells (*P* < .05; Figure 3D). All in all, these results indicated that knockdown of LINC00673 could repress paclitaxel and docetaxel resistance in prostate cancer cells.

### 3.4 LINC00673 silencing reduces methylation of the KLF4 gene promoter to up‐regulate KLF4 expression

**Figure 4:**
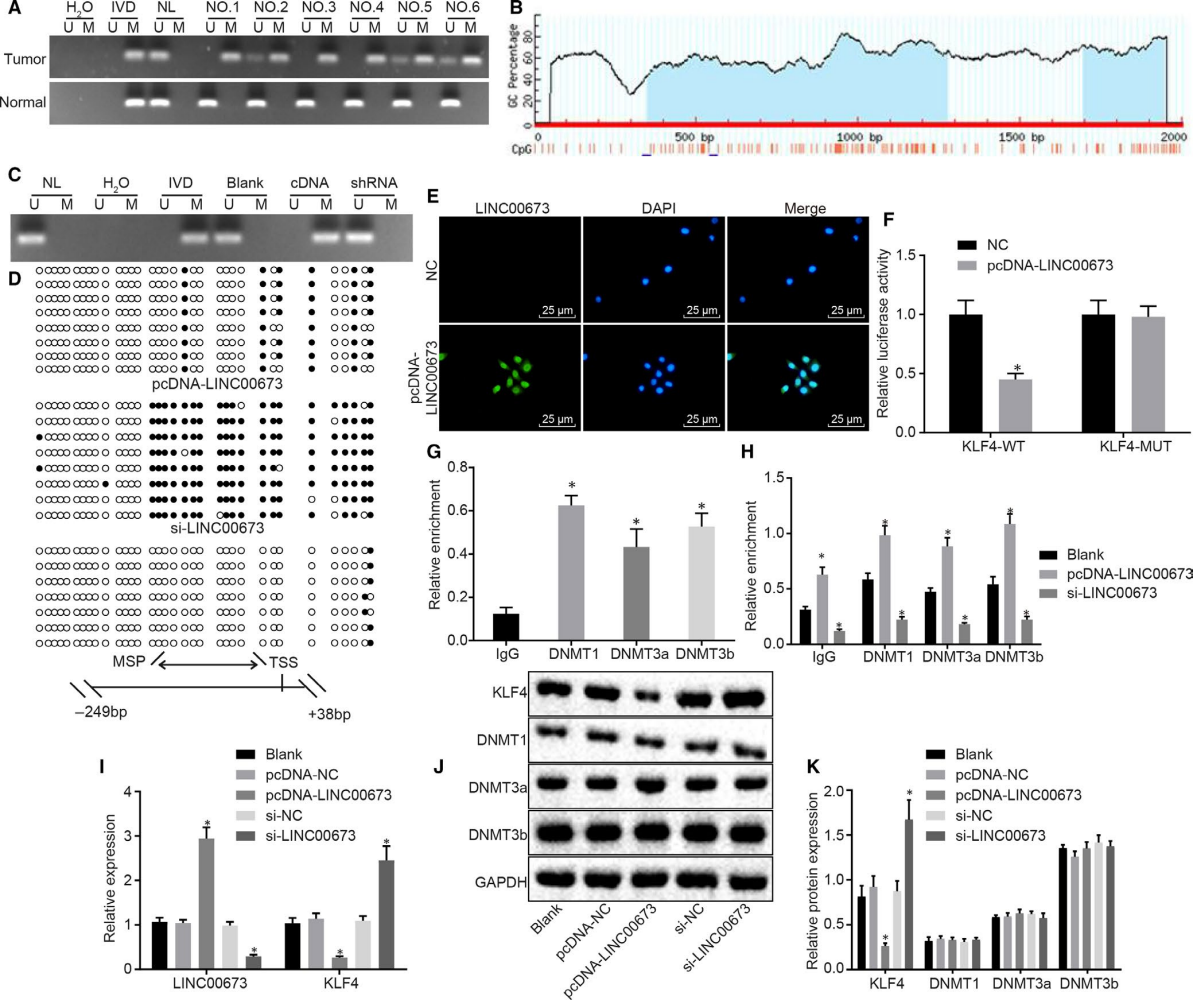
LINC00673 silencing inhibits KLF4 gene promoter methylation and further increases the expression of KLF4. A, Methylation of KLF4 gene promoter in prostate cancer tissues and adjacent normal tissues detected using MSP. B, Methylation of CpG islands in KLF4 gene promoter. C, Methylation of KLF4 gene promoter in DU145 cells treated with si‐LINC00673 or pcDNA‐LINC00673, as measured using MSP (H2O used as a dual negative control; In vitro methylated DNA (IVD) used as a positive methylation control; Normal lymphocyte DNA (NL) served as unmethylated positive control; U, unmethylation; M, methylation). D, Methylation of KLF4 gene promoter in DU145 cells treated with si‐LINC00673 or pcDNA‐LINC00673, as measured using BSP (double arrow, MSP amplification region; horizontal line, vulcanization sequence region; black circle, methylation site; white circle, unmethylation site; TSS, transcription start site; MSP, DNA methylation). E, Subcellular location of LINC00673 analysed using FISH (×400). F, Binding of LINC00673 to KLF4 verified using dual‐luciferase reporter gene assay. G, Enrichment of DNMT1, DNMT3a and DNMT3b in KLF4 gene promote region analysed using ChIP. H, Enrichment of DNMT1, DNMT3a and DNMT3b in KLF4 gene promote region in DU145 cells treated with si‐LINC00673 or pcDNA‐LINC00673 detected using RIP assay. I, LINC00673 expression and mRNA expression of KLF4 in DU145 cells following treatment of si‐LINC00673 or pcDNA‐LINC00673, as measured using RT‐qPCR. J, K, Western blot analysis of KLF4, DNMT1, DNMT3a and DNMT3b proteins in DU145 cells following treatment of si‐LINC00673 or pcDNA‐LINC00673. *P < .05 vs DU145 cells without treatment. Data (mean ± SD) among multiple groups were compared using one‐way ANOVA. The experiment was repeated in triplicate to obtain the mean value

Methylation specific PCR was performed to identify methylation of the KLF4 gene promoter in prostate cancer tissues, and it was found that KLF4 was highly methylated in prostate cancer tissues compared with adjacent normal tissues (*P* < .05; Figure 4A). In order to further verify the changes in the KLF4 gene promoter, the CpG islands in the KLF4 gene promoter were predicted using an online tool EMBOSS CpGPlot available at http://emboss.bioinformatics.nl/cgi-bin/emboss/cpgplot (Figure 4B). Moreover, methylation of CpG islands in the KLF4 gene promoter was detected using MSP and BSP, which revealed that CpG islands in the KLF4 gene promoter were highly methylated in DU145 cells treated with pcDNA‐LINC00673, whereas poor methylation was observed in DU145 cells treated with si‐LINC00673 (Figure 4C,D), suggesting that methylation of CpG islands in the KLF4 gene promoter was closely associated with the expression of LINC00673.

Moreover, FISH results revealed that LINC00673 was primarily located in the nucleus (Figure 4E). In addition, findings from dual‐luciferase reporter gene assay showed that the luciferase activity was attenuated in response to the co‐treatment of KLF4‐Wt and pcDNA‐LINC00673 (*P* < .05), but did not differ after co‐treatment with KLF4‐Mut and pcDNA‐LINC00673 (*P* > .05; Figure 4F). This finding suggested that LINC00673 could bind to KLF4 gene promoter region, which was consistent with the results predicted by a bioinformatics website. In order to further verify the binding of LINC00673 to the KLF4 gene promoter region, we subjected the KLF4 gene promoter in DU145 cells to ChIP analysis, the results of which illustrated that KLF4 gene promoter presented with significant enrichment of DNMT1, DNMT3a and DNMT3b (Figure 4G). Additionally, RIP assay was performed to analyse the binding between LINC00673 and the DNMTs with the DU145 cells treated with si‐LINC00673 or pcDNA‐LINC00673, and the results revealed that when compared with DU145 cells without treatment, LINC00673 was highly bound to DNMT1, DNMT3a and DNMT3b in DU145 cells treated with pcDNA‐LINC00673 (*P* < .05), but poorly bound to DNMT1, DNMT3a and DNMT3b in DU145 cells treated with si‐LINC00673 (*P* < .05; Figure 4H). Further, the effects of LINC00673 on the expression of KLF4 in DU145 cells were determined using RT‐qPCR and Western blot analysis. It was found that the expression of LINC00673 was up‐regulated, while that of KLF4 in DU145 cells was down‐regulated in response to pcDNA‐LINC00673 (*P* < .05); however, contrasting results were observed in the aforementioned factors in response to si‐LINC00673 (*P* < .05) when compared with the DU145 cells without treatment (Figure 4I‐K). In addition, Western blot analysis results also demonstrated that neither knockdown nor overexpression of LINC00673 exerted significant effects on the protein expressions of DNMT1, DNMT3a and DNMT3b (*P* > .05). These findings demonstrated that LINC00673 could drive methylation of the KLF4 gene promoter, and thus reduce the expression of KLF4 by recruiting DNMT1, DNMT3a and DNMT3b to the KLF4 gene promoter region.

### 3.5 LINC00673 silencing diminishes methylation of KLF4 gene promoter to suppress prostate cancer cell proliferation

**Figure 5:**
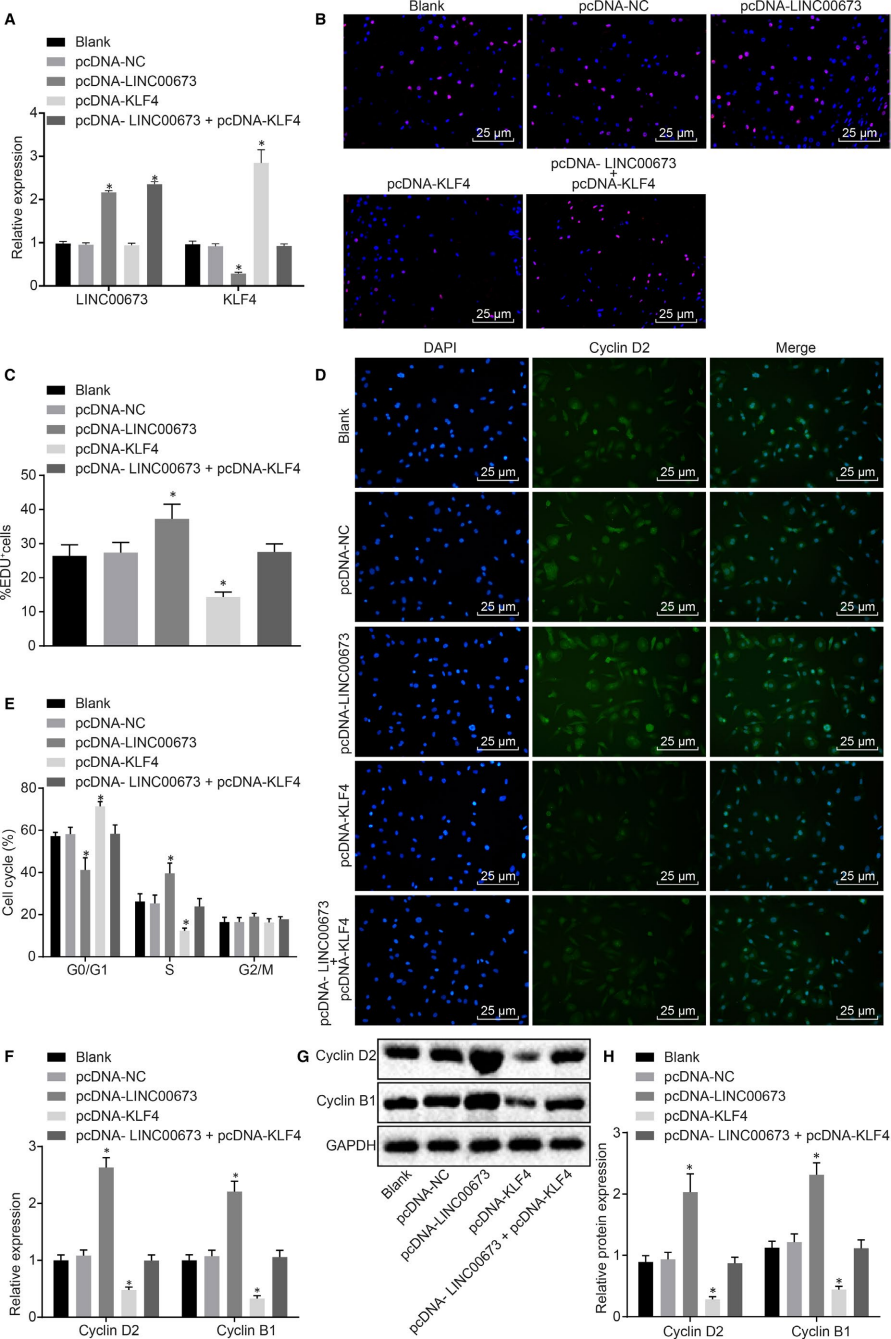
LINC00673 silencing inhibits prostate cancer cell proliferation by impairing the methylation of the KLF4 gene promoter. A, The transfection efficiency of overexpressed LINC00673 and KLF4 in cell lines detected by RT‐qPCR. B, C, Cell proliferation of DU145 cells treated with pcDNA‐KLF4, pcDNA‐LINC00673 or both detected by EdU assay (×400). D, Cell cycle of DU145 cells treated with pcDNA‐KLF4, pcDNA‐LINC00673 or both detected by flow cytometry. E, Cyclin D2 expression in DU145 cells treated with pcDNA‐KLF4, pcDNA‐LINC00673 or both detected by immunofluorescence staining (×400). F, mRNA expression of Cyclin D2 and Cyclin B1 in DU145 cells treated with pcDNA‐KLF4, pcDNA‐LINC00673 or both determined using RT‐qPCR. G and H, Western blot analysis of Cyclin D2 and Cyclin B1 proteins in DU145 cells treated with pcDNA‐KLF4, pcDNA‐LINC00673 or both. * *P* < .05 vs DU145 cells without treatment. Data (mean ± SD) among multiple groups were compared using one‐way ANOVA. The experiment was repeated in triplicate to obtain the mean value

Having identified the correlation between LINC00673 and KLF4, we moved to detect the transfection efficiency of overexpressed LINC00673 and KLF4 in cell lines using RT‐qPCR. The results showed that (Figure 5A) the expression of KLF4 was increased in DU145 cells treated with pcDNA‐KLF4 (*P* < .05), while that of KLF4 was decreased in DU145 cells treated with pcDNA‐LINC00673 when compared with DU145 cells without treatment (*P* < .05). No significant differences were observed in DU145 cells treated with pcDNA‐NC and pcDNA‐LINC00673 + pcDNA‐KLF4 (*P* > .05). Next, we shifted our focus on elucidating the mechanism by which LINC00673 influenced prostate cancer cell proliferation by regulating KLF4 methylation. EdU assay (Figure 5B,C) results revealed that EdU positive cells were stained with red colouration. In comparison with the DU145 cells without treatment, proliferative rates were attenuated in DU145 cells treated with pcDNA‐KLF4 (*P* < .05), while elevated rates were observed in DU145 cells treated with pcDNA‐LINC00673 (*P* < .05), but did not show any differences in DU145 cells treated with pcDNA‐NC and DU145 cells treated with both pcDNA‐LINC00673 and pcDNA‐KLF4 (both *P* > .05). In addition, the results from flow cytometry (Figure 5D) suggested that compared with DU145 cells without treatment, pcDNA‐KLF4‐treated DU145 cells presented with elevated proportion arrested at the G0/G1 phase, reduced proportion arrested at the S phase (*P* < .05), which was opposite to the trends observed in pcDNA‐LINC00673‐treated DU145 cells (*P* < .05); however, the cell cycle of DU145 cells presented with no significant differences in response to pcDNA‐NC treatment and combined treatment of pcDNA‐LINC00673 and pcDNA‐KLF4 (both *P* > .05). As shown by immunofluorescence staining (Figure 5E), Cyclin D2 in the nucleus was stained with green fluorescence. In contrast to cells without treatment, the fluorescence intensity of Cyclin D2 was found to be attenuated in pcDNA‐KLF4‐treated DU145 cells but potentiated in pcDNA‐LINC00673‐treated DU145 cells; the fluorescence intensity of Cyclin D2 did not differ in DU145 cells following pcDNA‐NC treatment and dual treatment of pcDNA‐LINC00673 and pcDNA‐KLF4 (both *P* > .05). Moreover, the mRNA and protein expression of Cyclin D2 and Cyclin B1 in DU145 cells were determined by RT‐qPCR and Western blot analysis. It was shown that pcDNA‐KLF4 treatment decreased the expressions of Cyclin D2 and Cyclin B1 (*P* < .05), while pcDNA‐LINC00673 treatment brought about the opposite results (*P* < .05); the expressions of Cyclin D2 and Cyclin B1 did not differ in response to pcDNA‐NC treatment and co‐treatment of pcDNA‐LINC00673 and pcDNA‐KLF4 (both *P* > .05; Figure 5F‐H). Taken together, these findings suggested that LINC00673 knockdown could repress prostate cancer cell proliferation by attenuating methylation of the KLF4 gene promoter.

### 3.6 LINC00673 silencing reduces the methylation of KLF4 gene promoter to suppress drug resistance in prostate cancer cells

**Figure 6:**
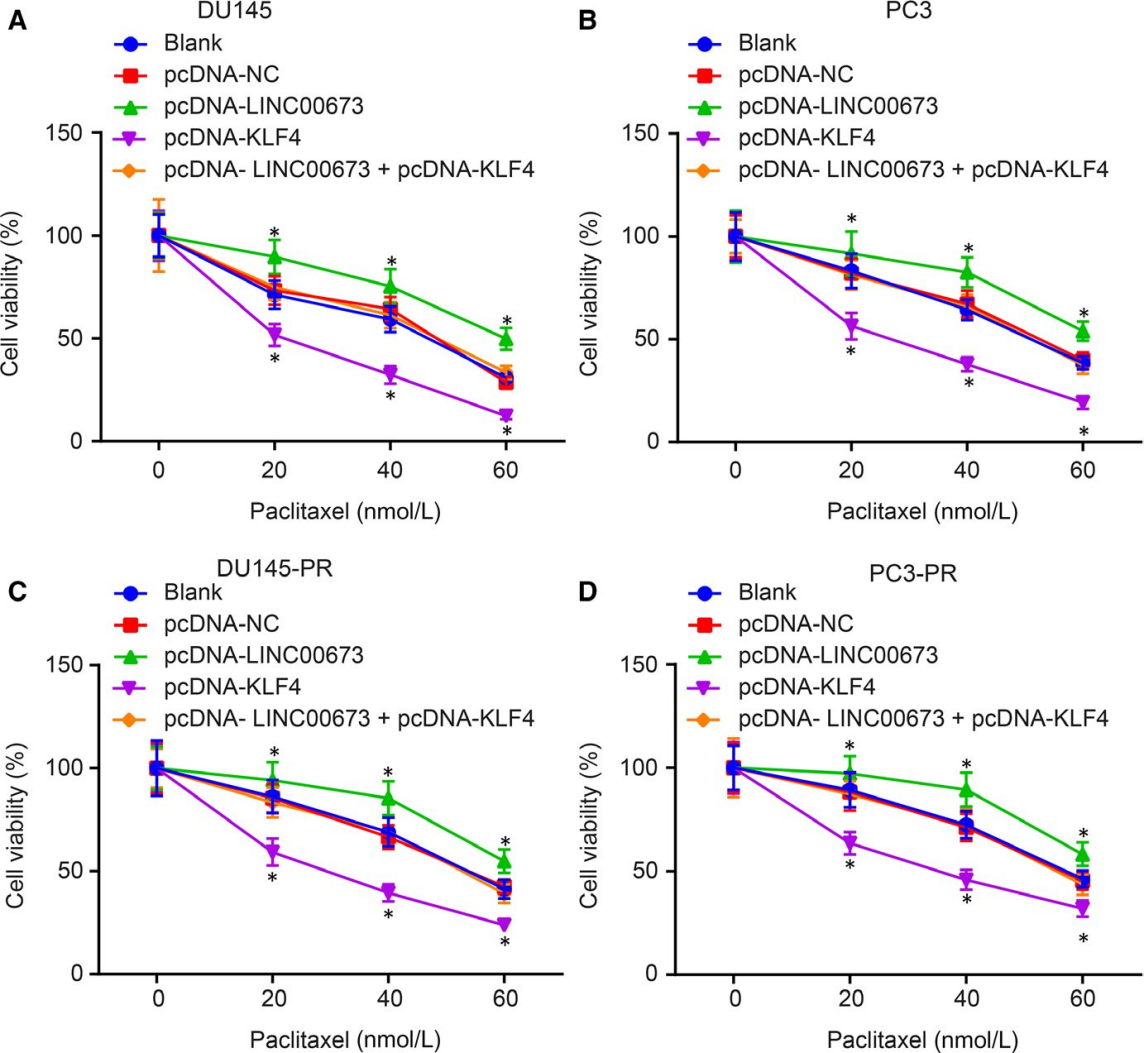
LINC00673 silencing decreases methylation of KLF4 gene promoter and further suppresses drug resistance of prostate cancer cells. A, DU145 cell proliferation following treatment with pcDNA‐KLF4, pcDNA‐LINC00673 or both determined using MTT assay. B, PC3 cell proliferation following treatment with pcDNA‐KLF4, pcDNA‐LINC00673 or both determined using MTT assay. C, Proliferation of paclitaxel‐resistant DU145 cells treated with pcDNA‐KLF4, pcDNA‐LINC00673 or both determined using MTT assay. D, Proliferation of paclitaxel‐resistant PC3 cells treated with pcDNA‐KLF4, pcDNA‐LINC00673 or both determined using MTT assay. *P < .05, vs DU145 and PC3 cells without treatment. Data (mean ± SD) among multiple groups were compared using repeated measures ANOVA. The experiment was repeated in triplicate to obtain the mean value

After determining the role of LINC00673 in prostate cancer cell proliferation and cell cycle, the focus of the experiment turned to elucidating the effects of LINC00673 on drug resistance in prostate cancer cells by regulating methylation of the KLF4 gene promoter with DU145 cells receiving treatment of pcDNA‐KLF4, pcDNA‐LINC00673 or both. An MTT assay was performed to measure the proliferation ability of DU145 cells. As shown in Figure 6A,C, after 48 hours of culture and treatment of paclitaxel at different concentrations, cell viability was elevated in DU145 cells treated with pcDNA‐LINC00673 (*P* < .05) and reduced in DU145 cells treated with pcDNA‐KLF4 (*P* < .05), with no obvious differences detected in pcDNA‐NC‐treated and pcDNA‐LINC00673 + pcDNA‐KLF4‐treated DU145 cells (*P* > .05) when compared with DU145 cells without treatment. Additionally, we repeated all the above‐mentioned experiments in PC3 cell lines. The results revealed that all the results obtained from PC3 cell lines were consistent with those from DU145 cell lines (Figure 6B,D). In summary, these findings solidified that LINC00673 could inhibit drug resistance in prostate cancer cells by reducing methylation of the KLF4 gene promoter.

### 3.7 LINC00673 silencing curtails methylation of KLF4 gene promoter to suppress the development of prostate cancer in vivo

**Figure 7:**
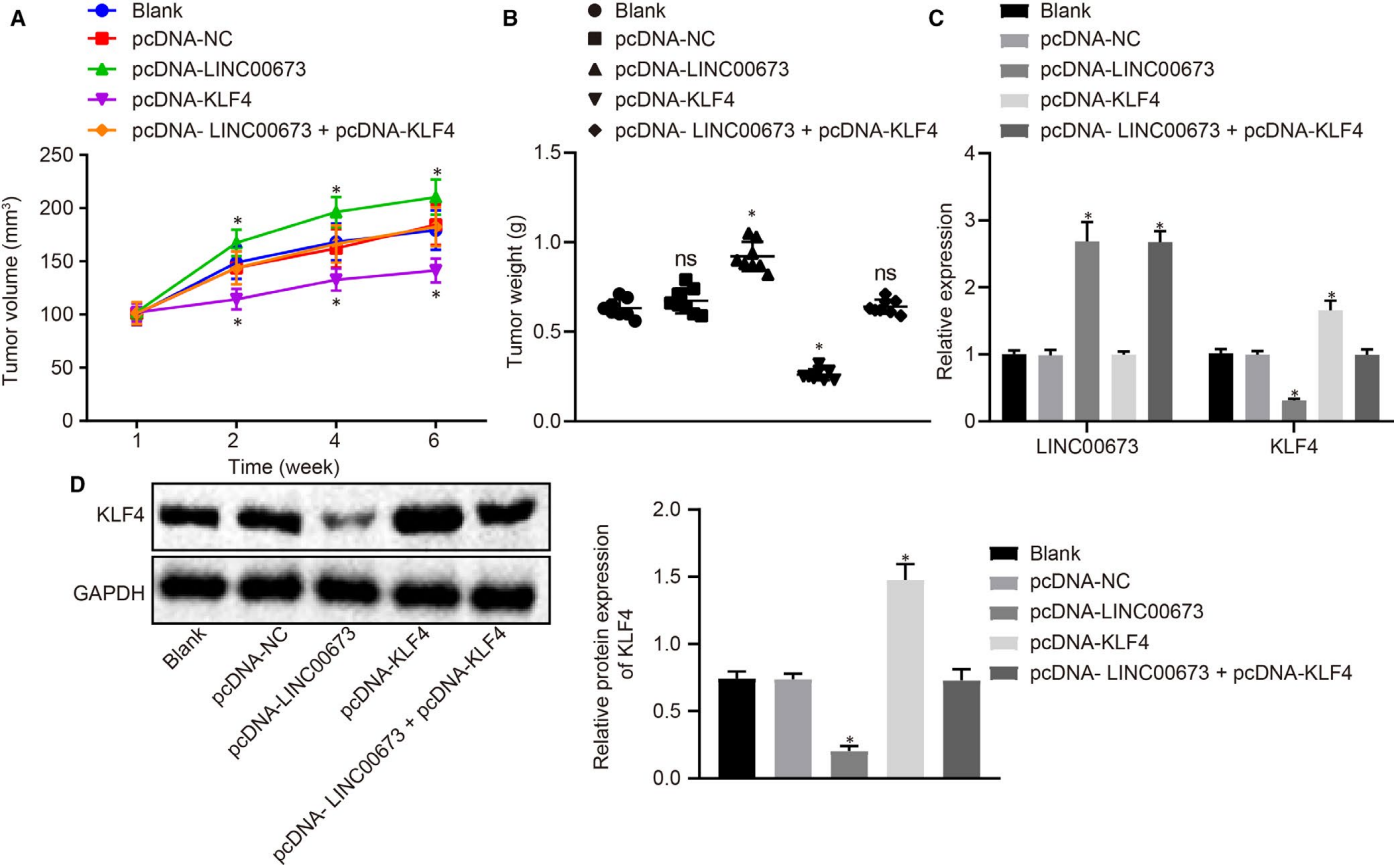
LINC00673 knockdown reduces methylation of KLF4 gene promoter and consequently retards the development of prostate cancer in vivo. A, B, xenograft tumours and quantitative analysis of tumour volume and weight after pcDNA‐KLF4, pcDNA‐LINC00673 or both treatments. C, LINC00673 expression and mRNA expression of KLF4 in tumour tissues after pcDNA‐KLF4, pcDNA‐LINC00673 or both treatments detected by RT‐qPCR. D, Western blot analysis of KLF4 protein in tumour tissues after pcDNA‐KLF4, pcDNA‐LINC00673 or both treatments. * *P* < .05, vs DU145 cells without treatment. Data (mean ± SD) among multiple groups were compared using one‐way ANOVA. N = 8

After treatment of pcDNA‐KLF4, pcDNA‐LINC00673 or both, DU145 cells were injected into the nude mice in order to establish subcutaneous xenograft tumour models. After 3 weeks of culture, the tumour radii were measured and recorded twice a week. The mice injected with pcDNA‐KLF4‐treated DU145 cells presented with reduced tumour volume (*P* < .05) while the mice injected with pcDNA‐LINC00673‐treated DU145 cells exhibited elevated tumour volumes (*P* < .05) when compared with mice injected with untreated DU145 cells; however, the tumour volume did not differ in mice injected with pcDNA‐NC‐treated and pcDNA‐LINC00673 + pcDNA‐KLF4‐treated DU145 cells (*P* > .05). After 3 weeks, the nude mice were intraperitoneally injected with paclitaxel (50 μg/kg), and the tumour volume was measured twice a week. The results showed that in contrast to injection of untreated DU145 cells, the tumour volume was slowly elevated following the injection of pcDNA‐KLF4‐treated DU145 cells, while quickly increased in response to the injection of pcDNA‐LINC00673‐treated DU145 cells; there were no tumour volume differences following injections of pcDNA‐NC‐treated and pcDNA‐LINC00673 + pcDNA‐KLF4‐treated DU145 cells (Figure 7A). Similar trends were also observed in regard to tumour weight. As depicted in Figure 7B, compared with mice without any treatment, the tumour weight was reduced in mice treated with pcDNA‐KLF4, while being elevated in mice treated with pcDNA‐LINC00673, whereas no evident differences were observed in mice treated with pcDNA‐LINC00673 + pcDNA‐KLF4 and pcDNA‐NC. RT‐qPCR was then conducted to detect the mRNA expression of KLF4 and LINC00673 in tumours, and Western blot analysis was conducted to determine KLF4 protein expression. The results illustrated in Figure 7C,D showed that mice treated with pcDNA‐LINC00673 + pcDNA‐KLF4 and pcDNA‐NC exhibited no remarkable changes in regard to KLF4 expression compared with mice without any treatment. However, pcDNA‐LINC00673 treatment resulted in diminished KLF4 expression yet elevated LINC00673 expression, while pcDNA‐KLF4 brought about increased KLF4 expression and pcDNA‐LINC00673 + pcDNA‐KLF4 led to elevated LINC00673 expression as compared with mice without any treatment. The aforementioned findings suggested that LINC00673 silencing could suppress the development and drug resistance of prostate cancer by attenuating methylation of the KLF4 gene promoter.

## 4 DISCUSSION

Prostate cancer is a highly prevalent cancer accompanied by high fatality rates and accounts for a substantial global burden on medical facilities and patients.^18^ Due to its variable natural history, researchers are confronted with various challenges in terms of optimum treatment of this heterogenous cancer.^1^ Therefore, it is imperative to seek new targets for the management of this disease to raise the quality of life of patients suffering from prostate cancer. Initial microarray‐based analyses indicated that both LINC00673 and KLF4 are differentially expressed in prostate cancer tissues. Accordingly, we performed in vivo and in vitro assays in the current study aiming to explore how LINC00673 influences the development of prostate cancer *via* regulation of KLF4. At last, our findings demonstrated that LINC00673 silencing could inhibit proliferation and drug resistance of prostate cancer cells via suppression of KLF4 gene promoter methylation.

Originally, we uncovered that LINC00673 was highly expressed while KLF4 was poorly expressed in both prostate cancer tissues and cell lines. Similarly, growing number of studies have documented high expression of LINC00673 in cancer tissues, for instance, LINC00673 was highly expressed in non‐small cell lung cancer (NSCLC) tissues when compared with normal lung tissues.^19^, ^20^ In addition, another study found that glioma cells exhibit up‐regulated LINC00673 expressions, and further highlighted down‐regulation of LINC00673 as an underlying biomarker for the management of metastatic glioma owing to its inhibitory role in the migration and invasion of glioma cells.^21^ Besides, multiple lncRNAs such as prostate cancer‐associated ncRNA transcript (PCAT)18 and ANRIL have also been previously found to be up‐regulated in prostate cancer tissues, and their expression was closely correlated with the biological progression of prostate cancer.^22^, ^23^ Interestingly, another key focus of the current study, the KLF4 gene, plays critical roles in cell growth, proliferation and differentiation, and has been further reported to potentially act as a tumour suppressor in prostate cancer owing to its decreased expression, which could be used to predict the prognosis of this cancer.^24^ Furthermore, depleted expressions of KLF4 have been detected in other cancers, such as breast cancer,^25^ while another study reported that overexpression of KLF4 could repress the growth and invasion of cancer cell lines, including cell lines of prostate cancer.^26^

Additionally, our findings demonstrated that LINC00673 could augment the methylation of the KLF4 gene promoter to consequently down‐regulate the expression of KLF4. This specific process of abnormal DNA methylation is associated with gene expression,^27^ and another study also noted that the expression of KLF4 could be depleted by promoter methylation in classical Hodgkin lymphoma cells.^28^ In line with our findings, Xu et al further demonstrated that KLF4 was poorly expressed, while the CpG islands were highly methylated in the KLF4 gene promoter in urothelial bladder cancer tissues.^29^ Furthermore, lncRNA PVT1, which was elevated in prostate cancer, was reported to serve as an oncogene in prostate cancer by promoting the methylation of microRNA‐164a.^30^ Consistently, results obtained from RIP and ChIP assays in a previously conducted study by Ba et al further proved that LINC00673 could diminish the expression of KLF4 *via* interaction with EZH2 and DNMT1.^9^

More importantly, the current study verified that LINC00673 silencing could suppress the proliferation and drug resistance of prostate cancer cells by attenuating the methylation of the KLF4 gene promoter. Lu et al demonstrated that proliferation and invasion abilities in NSCLC were suppressed following LINC00673 knockdown, suggesting that LINC00673 acts as an oncogene in NSCLC,^31^ which is line with the results attained in the current study. Similarly, LINC00673 silencing also exerts suppressive effects on cell invasion and migration in TSCC, which proposed LINC00673 as a promising prognostic and therapeutic target for TSCC.^7^ As reported by Siu et al, KLF4 serves as a tumour suppressor in prostate cancer, and its overexpression attenuates the cell proliferation and invasion abilities in prostate cancer independent of androgens.^32^ Moreover, KLF4 depletion induced by highly methylated KLF4 gene promoter has also been previously demonstrated to enhance the development of urothelial cancer, positioning KLF4 as a tumour suppressor; down‐regulated KLF4 or highly methylated KLF4 gene promoter could be considered as markers to predict the early recurrence of cancer.^33^ Drug resistance forms an enormous challenge for the management of advanced prostate cancer, and specifically, paclitaxel resistance in prostate cancer can potentially enhance cell migration via enforced extracellular matrix, leading to more invasive and aggressive phenotypes of this unfavourable cancer.^34^ Notably, KLF4 is also poorly expressed in breast cancer and has been further suggested as a possible protocol to reduce the resistance of breast cancer cells to tamoxifen.^35^ Similarly, lncRNA PCAT‐1 silencing attenuates drug resistance and invasion of colorectal cancer cells by reducing the expression of c‐Myc.^36^ Similarly, depletion of lncRNA plasmacytoma variant translocation 1, which was elevated in gastric cancer tissues and cells conferring resistance to cisplatin, could suppress the progression of multidrug resistance.^37^ Moreover, lncRNA MALAT1 down‐regulation was revealed to suppress cell proliferation and docetaxel chemoresistance in prostate cancer.^38^

**Figure 8:**
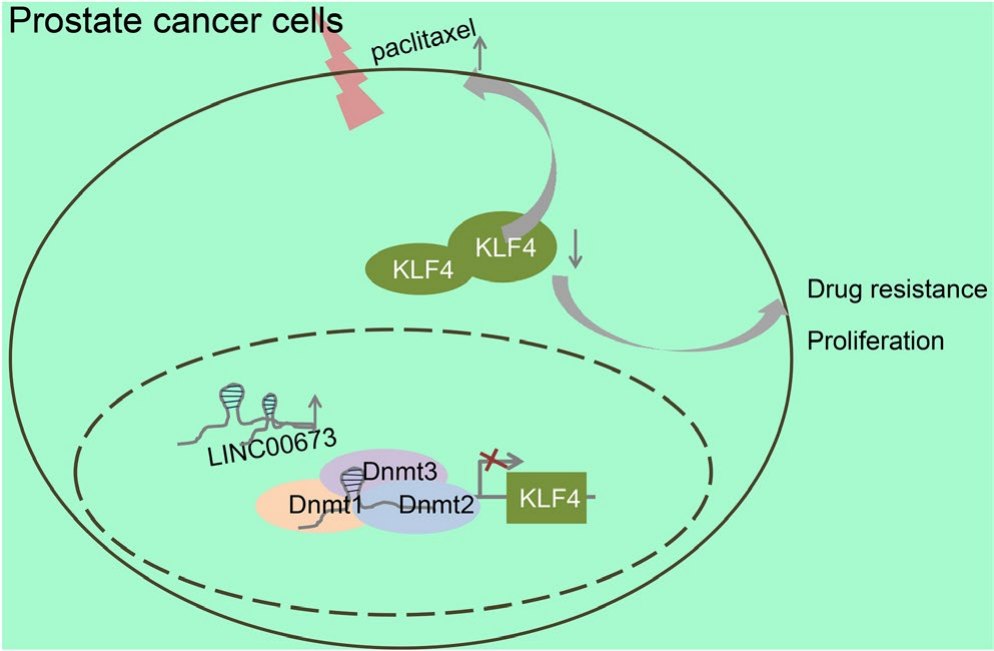
Regulatory mechanism of LINC00673 mediating proliferation and drug resistance of prostate cancer cells by regulating methylation of the KLF4 gene promoter. LINC00673 silencing decreases methylation of KLF4 gene promoter to up‐regulate the expression of KLF4, thus suppressing proliferation and drug resistance of prostate cancer cells

Taken together, our findings demonstrated that LINC00673 silencing could disrupt the methylation of the KLF4 gene promoter and consequently suppress cell proliferation and drug resistance in prostate cancer (Figure 8), illuminating potential for future therapeutic strategies for prostate cancer. For better management of prostate cancer, more efforts are warranted in order to uncover the molecular mechanism underlying the functions of LINC00673 in the development and progression of prostate cancer.

## CONFLICT OF INTEREST

The authors declare that they have no conflict of interest.

## AUTHOR CONTRIBUTIONS

Zhenming Jiang, Yuxi Zhang, Xi Chen, Pingeng Wu and Dong Chen designed the study. Zhenming Jiang, Xi Chen, Pingeng Wu, Dong Chen collated the data. Yuxi Zhang conceived and performed the experiments. Zhenming Jiang and Yuxi Zhang analysed the data and produced the initial draft of the manuscript. All authors contributed to the revision and approved the final submitted manuscript.

## ETHICS STATEMENT

The current study was approved by the Ethics Committee and Experimental Animal Ethics Committee of The First Hospital of China Medical University. Signed informed consents were obtained from all participants or their legal guardians prior to the experiment. All animal experimentation strictly adhered to principles aiming to minimize the number, suffering and discomfort of the included animals.

## CONSENT FOR PUBLICATION

Consent for publication was obtained from the participants.

## Data Availability

The data sets generated/analysed during the current study are available.
